# Lifestyle and medical conditions in relation to ALS risk and progression—an introduction to the Swedish ALSrisc Study

**DOI:** 10.1007/s00415-024-12496-w

**Published:** 2024-06-15

**Authors:** Charilaos Chourpiliadis, Christina Seitz, Anikó Lovik, Emily E. Joyce, Lu Pan, Yihan Hu, Ulf Kläppe, Kristin Samuelsson, Rayomand Press, Caroline Ingre, Fang Fang

**Affiliations:** 1 Institute of Environmental Medicine, Karolinska Institutet, Stockholm, Sweden; 2 Institute of Psychology, Leiden University, Leiden, The Netherlands; 3 Department of Clinical Neuroscience, Karolinska Institutet, Stockholm, Sweden; 4 Department of Neurology, Karolinska University Hospital, Stockholm, Sweden

**Keywords:** Amyotrophic lateral sclerosis, Motor neuron disease, Neuroepidemiology, Case-control study

## Abstract

**Background:**

This study was an introduction to the Swedish ALSrisc Study and explored the association of lifestyle and medical conditions, with risk and progression of amyotrophic lateral sclerosis (ALS).

**Methods:**

We included 265 newly diagnosed ALS patients during 2016–2022 in Stockholm and 207 ALS-free siblings and partners of the patients as controls. Information on body mass index (BMI), smoking, and history of head injuries, diabetes mellitus, hypercholesterolemia, and hypertension was obtained through the Euro-MOTOR questionnaire at recruitment. Patients were followed from diagnosis until death, invasive ventilation, or November 30, 2022.

**Results:**

Higher BMI at recruitment was associated with lower risk for ALS (OR 0.89, 95%CI 0.83–0.95), especially among those diagnosed after 65 years. One unit increase in the average BMI during the 3 decades before diagnosis was associated with a lower risk for ALS (OR 0.94, 95%CI 0.89–0.99). Diabetes was associated with lower risk of ALS (OR 0.38, 95%CI 0.16–0.90), while hypercholesterolemia was associated with higher risk of ALS (OR 2.10, 95%CI 1.13–3.90). Higher BMI at diagnosis was associated with lower risk of death (HR 0.91, 95%CI 0.84–0.98), while the highest level of smoking exposure (in pack-years) (HR 1.90, 95%CI 1.20–3.00), hypercholesterolemia (HR 1.84, 95%CI 1.06–3.19), and hypertension (HR 1.76, 95%CI 1.03–3.01) were associated with higher risk of death, following ALS diagnosis.

**Conclusions:**

Higher BMI and diabetes were associated with lower risk of ALS. Higher BMI was associated with lower risk of death, whereas smoking (especially in high pack-years), hypercholesterolemia, and hypertension were associated with higher risk of death after ALS diagnosis.

**Supplementary Information:**

The online version contains supplementary material available at 10.1007/s00415-024-12496-w.

## Introduction

Amyotrophic lateral sclerosis (ALS) is a relatively rare neurodegenerative disease with a poor prognosis [1]. Several mutations following either a monogenic, oligogenic, or polygenic inheritance have been associated with ALS, indicating a genetic component in the etiology of ALS [1]. Nevertheless, the heritability of the disease is estimated to be around 50% [2], suggesting that other factors, epigenetic or environmental, also contribute to the pathophysiology of ALS. The cause of ALS is hypothesized to involve genetic triggers of varying severity and their interaction with environmental risk factors through epigenomic changes at various time points during the lifespan [3]. The time of disease onset might depend on the mutation’s biological significance and the degree of exposure to environmental factors over time [4].

The most common monogenic mutation linked with ALS, especially in populations of European ancestry, is the repeat expansion of the GGGGCC hexanucleotide in the first intron of the *C9orf72* gene [5]. Repeats of 24 or more hexanucleotides are considered pathogenic, leading to earlier diagnosis of ALS [6, 7]. Nonetheless, monogenic ALS mutations may still exhibit incomplete penetrance or delayed onset [8], as exposure to certain environmental factors might modify the risk the mutations confer [8]. Many environmental risk factors have been studied in relation to ALS [9, 10], with some of the associations potentially varying by age, sex, and genetic factors [11–15].

The aim of this study was first to introduce the Swedish ALSrisc Study and then to explore the role of body mass index (BMI), smoking, and history of head injuries, hypercholesterolemia, diabetes mellitus, or hypertension on the risk and prognosis of ALS, focusing on the potential modifying effect of age, sex, and the mutation status of *C9orf72*.

## Methods

### ALSrisc study

**Fig. 1:**
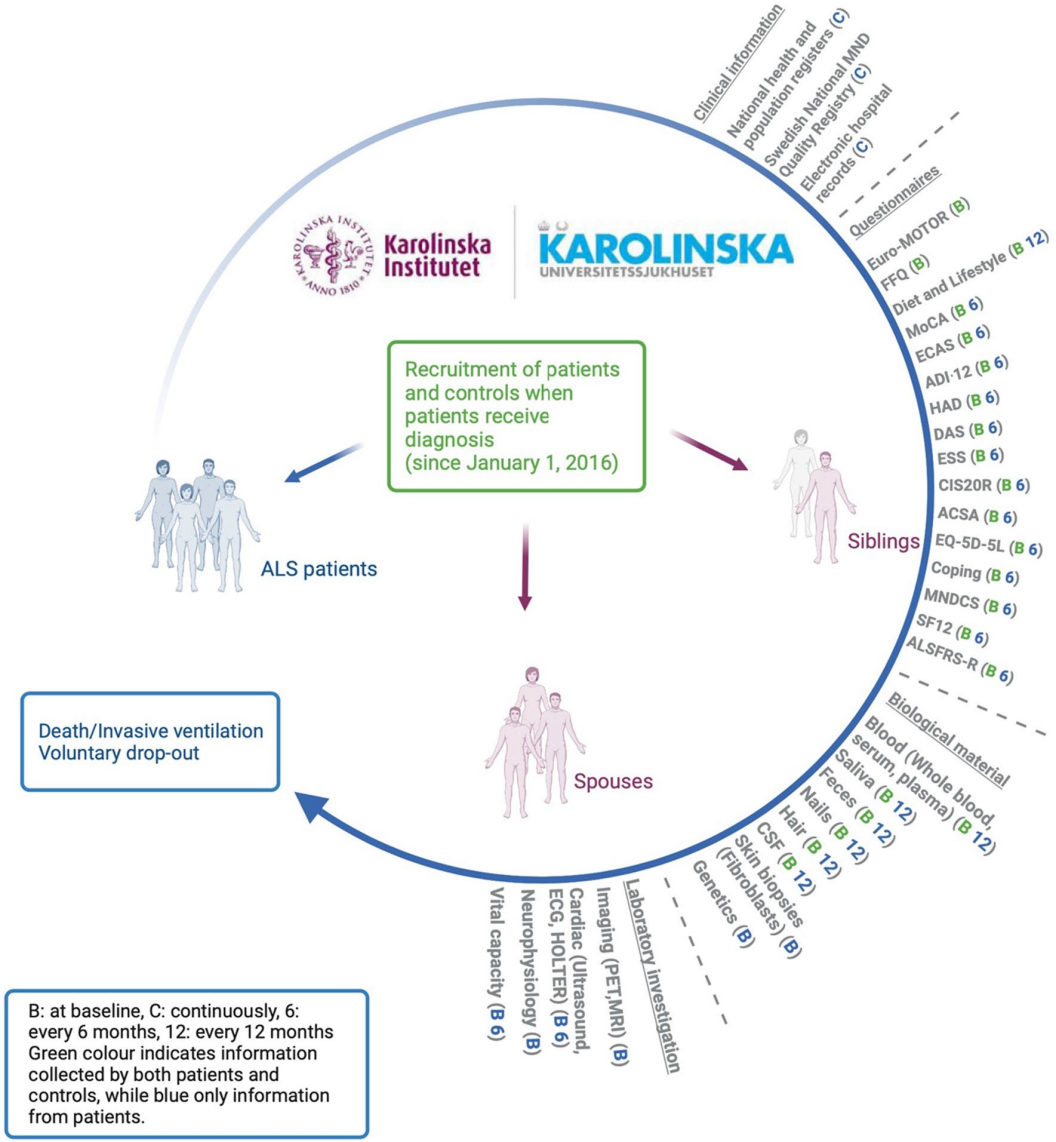
Overview of the ALSrisc Study. The central part of the figure illustrates the recruitment of patients and their relative controls (siblings and spouse) at the time of diagnosis of the patient or shortly thereafter. The circle represents the follow-up of the patients from diagnosis until death, initiation of invasive ventilation, or withdrawal from the study. A diverse set of information is collected, through linkage to the Swedish Motor Neuron Disease (MND) Quality Registry and electronic medical records, self-reported data from questionnaires, biological specimens, and laboratory investigations at recruitment or during follow-up (for patients only and at specific time intervals). The letter B indicates that information is collected at baseline (recruitment). The numbers “6” and “12” indicate that information is collected every 6 or 12 months. The letter C indicates that information is collected continuously. Blue indicates that information is collected only from ALS patients, whereas green indicates that information is collected from both patients and controls

**Fig. 2:**
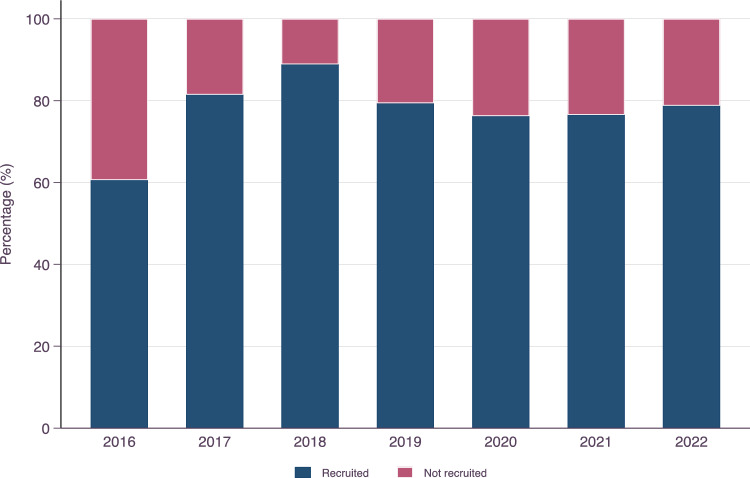
Recruitment rate in the ALSrisc Study

The ALSrisc Study (“Biomarkers and Risk Factors for Amyotrophic Lateral Sclerosis”) is an ongoing case–control study that started in 2016 in Stockholm, Sweden, with the overall goal of improving the understanding of ALS disease mechanisms by studying how specific biomarkers, lifestyle, and environmental factors are related to the risk and prognosis of ALS (see Fig. 1 for a graphical summary of the ALSrisc Study). Cases of the ALSrisc Study include all patients with a new diagnosis of ALS, progressive spinal muscular atrophy (PSMA), primary lateral sclerosis (PLS), or any other motor neuron diseases (MNDs) since January 2016. The patients are all diagnosed at the ALS Clinical Research Center, Karolinska University Hospital, the only tertiary center for ALS patients in the Stockholm region with a population of over 2 million. All patients with ALS met the revised El Escorial criteria for clinically definite, probable, or possible ALS. Furthermore, the ALSrisc Study includes two control groups, namely ALS-free full siblings and spouses of the cases. Cases, sibling controls, and spouse controls are enrolled at the time of diagnosis or shortly thereafter. Individuals not speaking Swedish and individuals who migrated out of the Stockholm region after recruitment (without complete follow-up) are excluded from the study. Figure 2 shows the recruitment rate of ALS patients by year in the ALSrisc Study.

Data collected in the study come from three major sources, namely medical records data, questionnaire data, and biological samples. Clinical characteristics of the ALS patients are collected from medical records, including time and site of disease onset, disease progression rate, spreading pattern of paresis, ALS Functional Rating Score-revised (ALSFRS-R), neurophysiology findings, medications, cognitive impairment including frontotemporal dementia (FTD), etc. Screening for a panel of 197 genes (e.g., *SOD1*, *TDP-43*, and *C9orf72*) is performed as clinical routine for ALS patients. In addition, patients are asked to participate in further examinations, including neuroimaging and cardiac examinations.

All patients included in the ALSrisc Study are automatically followed up through the Swedish MND Quality Registry [16]. Since 2015, this registry has continuously collected information on clinical measures, biological samples, and quality-of-life outcomes from the majority of the MND patients in Sweden at the time of diagnosis and each follow-up clinic visit every 3 months after diagnosis. Since 2017, this registry has collected information on nearly all MND patients in Stockholm. Through a structured interview using a questionnaire modified from a large European consortium for ALS “Euro-MOTOR”, we also collect self-reported information on previous environmental exposures (e.g., housing, employment, and military service), lifestyle factors (e.g., smoking, alcohol use, and physical activity), and medical history from the cases and controls [17]. We also use Food Frequency Questionnaires to collect information on past and current dietary habits from the cases and controls.

For ALS patients, we collect information at recruitment as well as every 6 months thereafter, on different measurements of mental health including ADI-12 (ALS Depression Inventory-12), HAD (Hospital Anxiety and Depression Scale), and DAS (Depression, Anxiety and Stress scale), cognitive function including MoCA (Montreal Cognitive Assessment) and ECAS (Edinburgh Cognitive and Behavioural ALS Screen ), quality of life including ESS (Epworth Sleepiness Scale), CIS20R (Checklist Individual Strength, Radbound University), ACSA (Amnestic Comparative Self-Assessment), EQ-5D-5L (EuroQoL 5-Dimensions 5-Levels), SF-12 (12-item Short Form Survey), MNDCS (Motor Neuron Disease Coping Scale), as well as Coping-AMS (Achievement Motives Scale), and the Lifestyle and Nutrition Survey.

All participants donate blood (stored as whole blood, plasma, and serum), feces, saliva, hair, and nails at enrollment. Feces, saliva, and blood samples are stored frozen at the Karolinska Institutet Biobank at −80 °C, whereas hair and nail samples are stored at room temperature. In addition, we collect cerebrospinal fluid (CSF) from the cases. Cases also donate these biological samples annually during the follow-up. To date, the ALSrisc Study has built up a large biobank including data and biospecimens collected from over 400 ALS patients and over 200 controls. The recruited ALS patients are largely representative of all ALS patients diagnosed during the same period in Stockholm, according to the Swedish MND Quality Registry (Table 1).

**Table 1:** Comparison of characteristics between ALS patients included in the ALSrisc Study and all ALS patients included in the Swedish Motor Neuron Disease (MND) Quality Registry diagnosed during March 2016 to May 2022 in Stockholm, Sweden

Characteristics	ALSrisc Study (*n* = 307)	MND Quality Registry (*n* = 389)
Age at diagnosis, median (IQR) [*N*]	67.13 (59.03–74.09) [307]	68.90 (60.27–74.90) [387]
Female, *N* (%)	144 (46.91%)	180 (46.27%)
BMI at diagnosis in Kg/m^2^, mean (SD) [*N*]	23.80 ± 3.97 [307]	23.81 ± 4.06 [387]
ALSFRS-R at diagnosis, median (SD) [*N*]	37.13 ± 7.33 [300]	36.18 ± 8.02 [355]
Progression rate at diagnosis, median (IQR) [*N*]	0.74 (0.39–1.38) [300]	0.78 (0.41–1.43) [353]
Diagnostic delay in months, median (IQR) [*N*]	12.01 (7.84–17.64) [300]	12.06 (7.65–18.33) [356]
Onset site, *N* (%)		
Spinal	179 (58.31%)	211 (54.24%)
Bulbar	110 (35.83%)	131 (33.68%)
Other	17 (5.54%)	21 (5.40%)
Missing	1 (0.33%)	26 (6.68%)
Invasive ventilation, *N* (%)	4 (1.30%)	5 (1.28%)
Number of deaths (mortality rate per 1000 person-years)	211 (441.4)	268 (461.6)
Median survival in years (IQR)	1.59 (0.85–2.94)	1.54 (0.79–2.95)
Familial disease, *N* (%)		
Familial	34 (11.07%)	34 (8.74%)
Sporadic	242 (78.83%)	242 (62.21%)
Unknown	30 (9.77%)	30 (7.71%)
Missing	1 (0.33%)	83 (21.34%)
Dementia, *N* (%)	23 (7.49%)	23 (5.91%)

*ALS* amyotrophic lateral sclerosis, *ALSFRS-R* amyotrophic lateral sclerosis functional rating scale-revised, *BMI* body mass index, *IQR* interquartile range, *SD* standard deviation

### Study material

In the present study, we included 265 patients with newly diagnosed ALS from March 2016 to May 2022 recruited to the ALSrisc Study and followed these patients from the date of diagnosis until the date of death, initiation of invasive ventilation, or the end of follow-up (November 30, 2022), whichever came first. The site of disease onset was classified as bulbar, spinal, or other (including respiratory and frontal lobe onset). The time to diagnosis was calculated as the time interval between the onset of muscle paresis and the diagnosis of ALS. The functional decline was calculated using the ALSFRS-R measured at diagnosis and during follow-up. The disease progression rate at diagnosis was calculated by the following formula: (48–ALSFRS-R score at diagnosis)/diagnostic delay (in months). Information on *C9orf72* mutation status was procured from the genetic panel mentioned above.

We included 207 controls (79 siblings and 128 partners of the ALS patients) in the present study. None of these siblings tested positive for *C9orf72* mutation and remained asymptomatic during the study. In addition to age and sex, self-reported information on BMI, smoking, and history of head injuries, diabetes mellitus, hypercholesterolemia, or hypertension was collected at recruitment for both cases and controls. BMI was estimated as the ratio of the current reported weight (kilograms) and height (m^2^) at recruitment, as well as for each decade before recruitment since the age of 20. Smoking status was classified as current, former, or never. We calculated the pack-years of smoking by multiplying the number of cigarettes smoked daily by the years of smoking. Study participants were then categorized into four groups: never smoked, smoked < 7 pack-years, smoked 7–16 pack-years, or smoked > 16 pack-years. The latter three groups were classified based on the tertile distribution of pack-years among the participants who smoked. Information on past medical history was collected to establish a previous diagnosis of diabetes mellitus, hypercholesterolemia, and hypertension. Self-reported information on the use of medications for the treatment of hypercholesterolemia and hypertension was used as an additional source to ascertain history of hypercholesterolemia and hypertension.

### Statistical analysis

We first used unconditional logistic regression models to calculate the odds ratio (OR) of ALS with 95% confidence interval (95% CI) in relation to BMI, smoking, and history of head injuries, diabetes mellitus, hypercholesterolemia, or hypertension, by comparing cases and controls. In the analysis of smoking, we examined the risk of ALS in relation to smoking status (never, former, or current) as well as by different pack-years of smoking (never smoked, or < 7, 7–16, or > 16 pack-years). Additionally, we assessed the non-linear association between BMI, modeled with restricted cubic splines with three knots at the 10th, 50th, and 90th percentiles, and ALS risk. As a secondary analysis, we used conditional logistic regression models with the family identifier as stratum to account for potential familial confounding due to shared early life environmental and genetic factors (between siblings) and adult-life environmental factors (between partners). In both analyses, we adjusted for age at recruitment and sex. For BMI during the 3 decades before recruitment, we also used generalized estimating equations (GEE) with independent correlation structure and robust standard errors to calculate the association of BMI reported for every decade until recruitment with the risk for ALS. The GEE model was adjusted for age and sex. Finally, we used the method of locally weighted scatterplot smoothing (LOWESS) to plot the mean BMI, separately for cases and controls, as well as for cases of different *C9orf72* mutation status, during each decade before recruitment.

Then, to study the association of BMI, smoking, and history of head injuries, diabetes mellitus, hypercholesterolemia, or hypertension with the time-varying ALSFRS-R score and the risk of death, a joint model that combines a linear mixed-effects model for all the repeated measurements of ALSFRS-R score and a survival model of censored outcomes was used. In the longitudinal component of the model, we considered a random intercept, a random slope, and an unstructured covariance matrix, while for the survival model, we considered a Royston–Parmar proportional hazards survival model where the baseline log hazard function was modeled with restricted cubic splines with four knots at the 5th, 35th, 65th, and 95th percentiles. The proportional hazards assumption was tested using scaled Schoenfeld residuals, and interactions with time were introduced for any variables violating this assumption. The attained age was used as the underlying time scale, and the date of birth was used as the time origin. The longitudinal and the survival components were linked through the shared random effects. The model was adjusted for age at diagnosis, sex, site of onset, diagnostic delay, and BMI at diagnosis. The latter was not included in models where the main independent variable was BMI. In secondary analyses, we also performed a Cox model and a linear mixed model to assess the associations between these factors and risk of death or longitudinal changes of ALSFRS-R after a diagnosis of ALS, respectively, after adjusting for age at diagnosis, sex, site of onset, diagnostic delay, and BMI at diagnosis. The survival model was additionally adjusted for ALSFRS-R and progression rate at the time of diagnosis.

Statistical analyses were performed using STATA (*Release 16*. College Station, TX: StataCorp LLC.). A predefined significance level of 5% was used for all the analyses. Reporting of the present study followed the STrengthening the Reporting of OBservational studies in Epidemiology (STROBE) guidelines (Supplementary File 1). The missingness level was low (5–10%) in variables with missing values; thus, we performed complete case analyses. Figure 1 was created using BioRender.com.

### Standard protocol approval, registration, and patient consent

The study was approved by the Swedish Ethical Review Authority (DNRs: 2014–1815-31–4, 2018–1605-31, and 2021–06397-02). Oral and written informed consent was collected from all study participants.

## Results

In the present study, 117 cases (44.3%) were women, whereas 116 controls were women (56%). The mean (SD) age at recruitment was 65.6 (10.6) for cases and 63.7 (10.7) for controls (Table 2, Supplementary Table 1). Among the cases, the mean (SD) ALSFRS-R score at diagnosis was 37.1 (7.6). Spinal onset was observed in 62.7% of the cases, and bulbar onset in 30.4%. The median (IQR) diagnostic delay was 13.4 (8.6–20.9) months.

**Table 2:** Baseline characteristics of ALS patients and ALS-free spouse and sibling controls—an analysis of the ALSrisc Study

Characteristics	Cases (*n* = 265)	Spouses (*n* = 128)	Siblings (*n* = 79)
Age at recruitment, mean (SD) [*N*]	65.6 (10.62) [265]	64.75 (10.88) [128]	61.92 (10.15) [79]
Female, *N* (%)	117 (44.15)	72 (56.25)	44 (55.70)
BMI at recruitment in Kg/m^2^, mean (SD) [*N*]	24.25 (3.73) [261]	25.74 (3.94) [126]	25.86 (5.07) [77]
BMI during the 10 years before recruitment, mean (SD) [*N*]	25.69 (4.08) [246]	25.15 (3.86) [125]	25.42 (5.43) [71]
BMI during the 20 years before recruitment, mean (SD) [*N*]	25.10 (4.03) [234]	24.37 (3.46) [121]	24.33 (4.49) [70]
BMI during the 30 years before recruitment, mean (SD) [*N*]	23.59 (3.08) [214]	23.48 (3.75) [109]	23.22 (4.1) [68]
BMI during the 40 years before recruitment, mean (SD) [*N*]	22.73 (3.46) [187]	22.09 (2.88) [91]	22.01 (3.76) [56]
Smoking status, *N* (%)			
Current smokers	23 (8.68)	12 (9.38)	6 (7.59)
Former smokers	121 (45.66)	67 (52.34)	29 (36.71)
Never smoked	121 (45.66)	49 (38.28)	44 (55.70)
Smoking in pack-years, *N* (%)			
Never smoked	121 (45.66)	49 (38.28)	44 (55.70)
< 7	33 (12.45)	32 (25.0)	9 (11.39)
7–16	42 (15.85)	18 (14.06)	12 (15.19)
> 16	45 (16.98)	17 (13.28)	7 (8.86)
History of head injuries, *N* (%)			
No	219 (82.64)	111 (86.72)	59 (74.68)
Yes	46 (17.36)	17 (13.28)	20 (25.32)
History of diabetes mellitus, *N* (%)			
No	239 (91.92)	109 (87.20)	69 (88.46)
Yes	21 (8.08)	16 (12.80)	9 (11.54)
History of hypertension, *N* (%)			
No	151 (62.40)	82 (68.33)	50 (68.49)
Yes	91 (37.60)	38 (31.67)	23 (31.51)
History of hypercholesterolemia, *N* (%)			
No	195 (75.29)	99 (79.20)	66 (85.71)
Yes	64 (24.71)	26 (20.80)	11 (14.29)
ALSFRS-R at diagnosis, median (SD) [*N*]	37.58 (7.29) [222]		
Progression rate at diagnosis, median (IQR) [*N]*	0.62 (0.32–1.29) [218]		
Diagnostic delay, median (IQR) [*N*]	13.4 (8.6–20.9) [261]		
Onset site, *N* (%)			
Other	19 (7.17)		
Spinal	163 (61.51)		
Bulbar	79 (29.81)		
Missing	4 (1.51)		

*ALS* amyotrophic lateral sclerosis, *ALSFRS-R* amyotrophic lateral sclerosis functional rating scale-revised, *BMI* body mass index, *IQR* interquartile range, *SD* standard deviation

### Cases versus controls

**Fig. 3:**
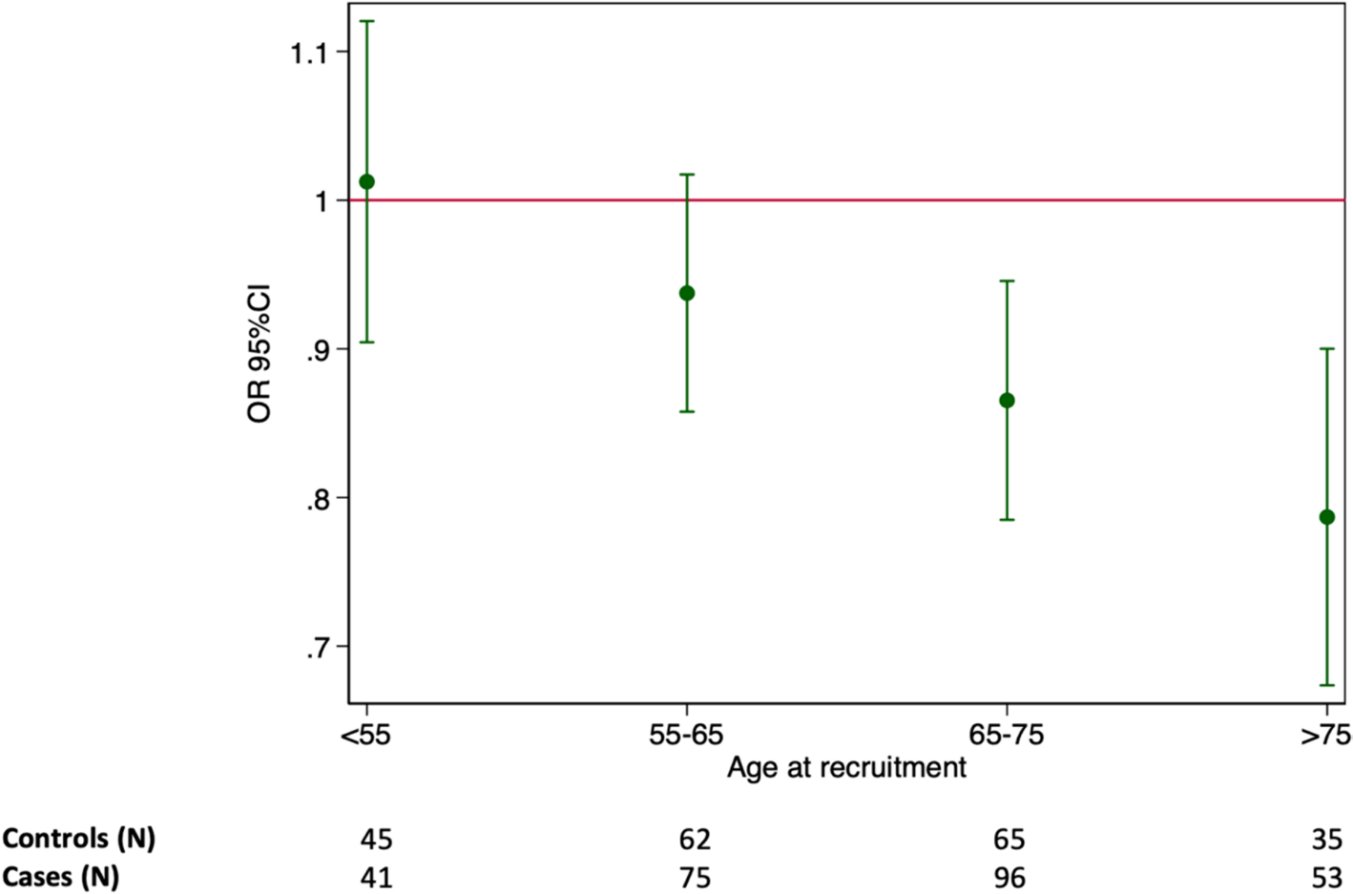
Odds ratio (OR) with 95% confidence interval (CI) for ALS per 1 kg/m2 increase in BMI measured at recruitment, adjusted for sex—stratified analysis by age at recruitment. Odds ratio (circle) with 95% confidence interval (line). The absolute numbers of cases and controls are shown in the bottom of the figure

**Fig. 4:**
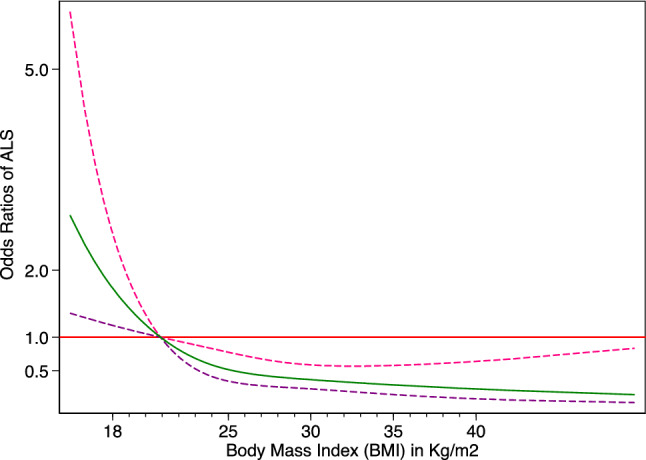
Odds ratio (OR) and 95% confidence interval (CI) for ALS in relation to BMI measured at recruitment, adjusted for sex analysis using BMI as a continuous variable. Odds ratio (solid line) with 95% confidence interval (dashed lines) for ALS in relation to BMI (Kg/m2) modeled with restricted cubic splines with three knots placed at a BMI of 20.7, 24.2, and 29.9, respectively. Estimates are derived from unconditional logistic regression with linear and spline terms for BMI and a BMI of 21 kg/m2 as the reference value

**Fig. 5:**
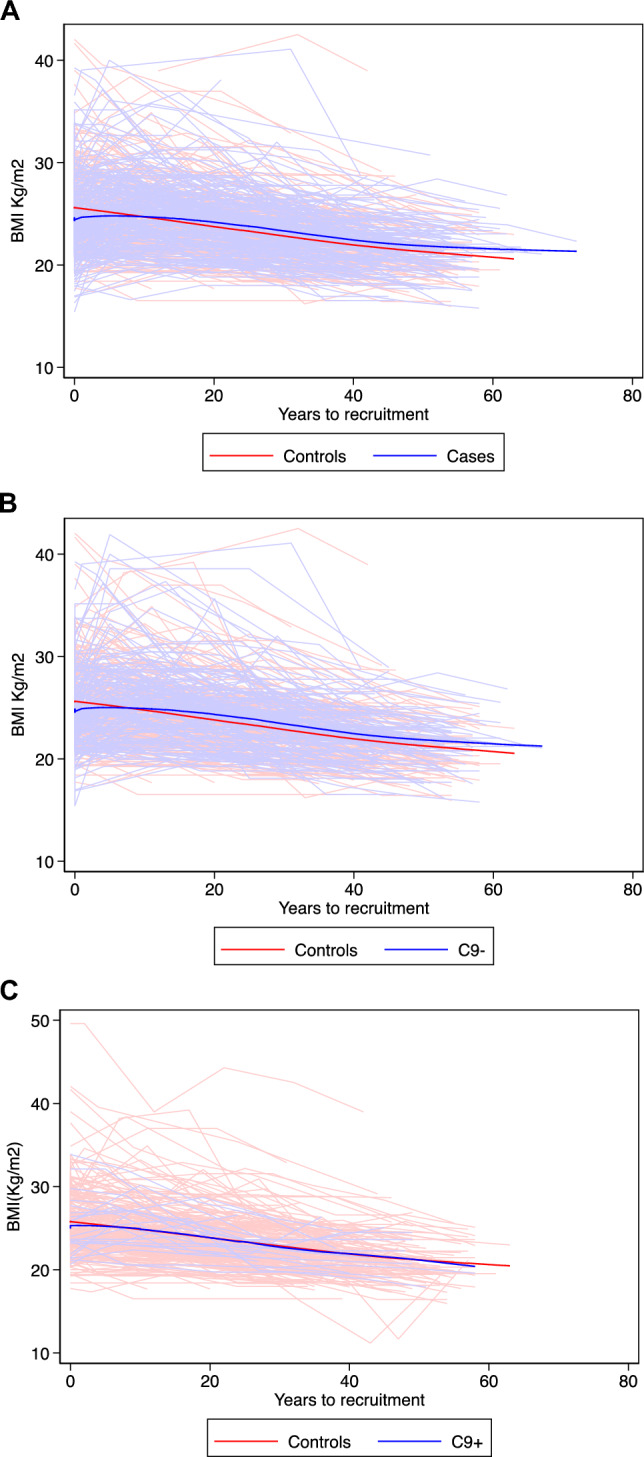
Mean BMI for each decade before recruitment among ALS patients and controls. **A** all patients and controls, **B** patients without the C9orf72 mutation and their controls, **C** patients with C9orf72 mutation and their controls

In the unconditional logistic regression, one-unit increase in BMI at recruitment was associated with 10% lower odds for ALS (OR 0.90, 95%CI 0.85–0.96) (Table 3). The analysis by age at recruitment showed that a statistically significant association was only observed among individuals 65 years or older (Fig. 3). At the time of recruitment, compared to a BMI of 21, a lower BMI was associated with higher odds of ALS, whereas a higher BMI was associated with lower odds (Fig. 4). The conditional logistic regression models yielded similar results, showing a slightly stronger association for BMI at recruitment, a positive association between hypercholesterolemia and ALS (OR 2.10, 95%CI 1.13–3.90), and an inverse association between diabetes mellitus and ALS (OR 0.38, 95%CI 0.16–0.89). After adjusting additionally for the earliest available BMI measurement which was on average 42.6 ± 11.7 years before diagnosis, we found similar associations for diabetes mellitus (OR 0.35, 95%CI 0.15–0.85) and hypercholesterolemia (OR 2.21, 95%CI 1.14–4.26). Although no association was noted between BMI during the three different decades before recruitment and the risk of ALS, we found an increasing BMI in both cases and controls by time to recruitment (Fig. 5). Although cases had a slightly higher BMI than controls earlier in life, they experienced a slower increase in BMI along with aging, resulting in lower BMI levels during the last decade before diagnosis. This trend was similar when comparing ALS patients without the *C9orf72* mutation (C9- cases) to controls. The difference was, however, negligible between ALS patients with the *C9orf72* mutation (C9 + cases) and controls. Table 4 shows the association of one-unit increase in BMI for every decade during the 3 decades before recruitment with the risk for ALS. Over time, one-unit increase in BMI was associated with 6% lower odds for ALS (OR 0.94, 95%CI 0.89–0.99). This association did not differ greatly by sex or C9 status.

**Table 3:** Adjusted odds ratio (OR) with 95% confidence interval (CI) of ALS in relation to BMI, smoking, and history of head injuries, diabetes mellitus, hypercholesterolemia, or hypertension—analysis using unconditional or conditional logistic regression model

Characteristics	No. of cases/ controls	Unconditional	Conditional
		OR (95% CI)	OR (95% CI)
BMI at recruitment in Kg/m^2^ (per 1 kg/m2)	146/193	**0.90 (0.85, 0.96)**	**0.89 (0.83, 0.95)**
BMI during the 10 years before recruitment (per 1 kg/m2)	137/186	1.00 (0.95, 1.06)	0.99 (0.93, 1.05)
BMI during the 20 years before recruitment (per 1 kg/m2)	130/181	1.03 (0.97, 1.10)	1.02 (0.95, 1.09)
BMI during the 30 years before recruitment (per 1 kg/m2)	120/167	1.01 (0.95, 1.08)	1.02 (0.94, 1.11)
BMI during the 40 years before recruitment (per 1 kg/m2)	104/137	1.05 (0.97, 1.14)	1.13 (0.99, 1.30)
Smoking status			
Current smokers	9/18	0.58 (0.24, 1.38)	0.41 (0.14, 1.18)
Former smokers	62/89	0.75 (0.47, 1.19)	0.71 (0.42, 1.21)
Never smoked	76/89	Ref	Ref
Smoking in pack-years			
< 7	18/37	0.55 (0.29, 1.06)	0.61 (0.30, 1.28)
7–16	22/28	0.80 (0.42, 1.55)	0.62 (0.29, 1.32)
> 16	22/23	1.02 (0.52, 2.01)	0.76 (0.32, 1.77)
Never smoked	76/89	Ref	Ref
History of head injuries			
Yes	25/35	0.92 (0.51, 1.65)	1.12 (0.58, 2.19)
No	124/161	Ref	Ref
History of diabetes mellitus			
Yes	10/24	**0.42 (0.19, 0.93)**	**0.38 (0.16, 0.90)**
No	135/168	Ref	Ref
History of hypercholesterolemia			
Yes	41/35	1.59 (0.93, 2.70)	**2.10 (1.13, 3.90)**
No	103/156	Ref	Ref
History of hypertension			
Yes	54/56	1.42 (0.87, 2.31)	1.35 (0.76, 2.41)
No	79/128	Ref	Ref

*ALS* amyotrophic lateral sclerosis, *BMI* body mass index, *Ref* reference

**Table 4:** Longitudinal analyses for BMI (per 1 kg/m^2^) up to three decades before diagnosis in relation to risk of ALS

Analysis	OR (95% CI)
Overall	0.94 (0.89, 0.99)
Stratified analysis by sex	
Female	0.96 (0.90, 1.04)
Male	0.92 (0.85, 0.99)
Separate analysis for C9 + and C9- patients	
C9 + cases vs controls	0.96 (0.87, 1.07)
C9- cases vs controls	0.95 (0.90, 1.01)

*ALS* amyotrophic lateral sclerosis, *BMI* body mass index, *CI* confidence interval, *C9+ or C9-* patients with or without the *C9orf72* mutation, *OR* odds ratio

### Analyses among cases

During a median (IQR) follow-up of 1.55 (0.95–2.45) years, we observed a total of 164 deaths or initiation of invasive ventilation among the 265 ALS patients. Table 5 shows the association of the studied factors with the risk of death following an ALS diagnosis, after accounting for the repeated measurements of ALSFRS-R after ALS diagnosis. Patients with a higher BMI at diagnosis had a lower risk of death (HR 0.91, 95%CI 0.84–0.98), while a history of hypercholesterolemia (HR 1.84, 95%CI 1.06–3.19) or hypertension (HR 1.76, 95%CI 1.03–3.01) was associated with a higher risk of death after ALS diagnosis. Current smoking (HR 2.28; 95%CI 0.91–5.71) and the highest level of smoking in pack-years (HR 1.90, 95%CI 1.20–3.00) were both associated with a higher risk of death, although the association was only statistically significant for the latter. No association was found for history of head injuries or diabetes mellitus. Similar estimates were found when studying the risk of death without accounting for the longitudinal change of ALSFRS-R (Supplementary Table 2). In the analysis of repeated measurements for ALSFRS-R alone, ALS patients with the highest level of smoking in pack-years (β −3.81, 95%CI −7.24 to −0.38) or a history of hypertension (β −4.55, 95%CI −7.22 to −1.89) or hypercholesterolemia (β −2.86, 95%CI −5.75 to 0.02) had a faster decline in ALSFRS-R compared with other patients (Supplementary Table 3).

**Table 5:** Adjusted hazard ratio (HR) with 95% confidence interval (CI) for risk of death after ALS diagnosis in relation to BMI, smoking, and history of head injuries, diabetes mellitus, hypercholesterolemia, or hypertension after accounting for the longitudinal changes of ALSFRS-R on the survival time—analysis using a joint longitudinal and survival model

Characteristics	N of events (IR)	HR (95% CI)
BMI at diagnosis (per 1 kg/m2)	151 (0.34)	**0.91 (0.84, 0.98)**
BMI during the 10 years before diagnosis (per 1 kg/m2)	143 (0.34)	0.95 (0.88, 1.02)
BMI during the 20 years before diagnosis (per 1 kg/m2)	136 (0.34)	0.94 (0.88, 1.01)
BMI during the 30 years before diagnosis (per 1 kg/m2)	126 (0.35)	0.89 (0.79, 1.00)
BMI during the 40 years before diagnosis (per 1 kg/m2)	113 (0.37)	0.92 (0.83, 1.02)
Smoking status		
Current smokers	13 (0.38)	2.28 (0.91, 5.71)
Former smokers	72 (0.42)	1.19 (0.71, 2.02)
Never smoked	66 (0.28)	Ref
Smoking in pack-years		
< 7	18 (0.36)	1.38 (0.80, 2.36)
7–16	27 (0.42)	1.21 (0.75, 1.95)
> 16	28 (0.56)	**1.90 (1.20, 3.00)**
Never smoked	66 (0.28)	Ref
History of head injuries		
Yes	23 (0.39)	1.33 (0.69, 2.56)
No	128 (0.34)	Ref
History of diabetes mellitus		
Yes	10 (0.29)	0.56 (0.20, 1.57)
No	137 (0.34)	Ref
History of hypercholesterolemia		
Yes	42 (0.40)	**1.84 (1.06, 3.19)**
No	105 (0.32)	Ref
History of hypertension		
Yes	62 (0.46)	**1.76 (1.03, 3.01)**
No	74 (0.27)	Ref

*ALS* amyotrophic lateral sclerosis, *ALSFRS-R* amyotrophic lateral sclerosis functional rating scale-revised, *BMI* body mass index, *Re*f reference

## Discussion

We investigated BMI, smoking, and history of head injuries, hypercholesterolemia, diabetes mellitus, or hypertension in relation to the risk of ALS in the Swedish ALSrisc Study. Among the ALS patients, we explored the association of these factors with the risk of death and functional decline after diagnosis. In brief, we found that ALS patients, primarily those above 65, had a lower BMI at the time of diagnosis, compared to controls. We further showed that a one-unit increase in the average BMI during the 30 years before diagnosis was associated with a 6% lower risk for ALS. Diabetes was also associated with a lower risk of ALS. A higher BMI at the time of diagnosis was associated with a lower risk of death, whereas smoking (especially in higher dose) and a history of hypertension or hypercholesterolemia were associated with a higher risk of death following an ALS diagnosis.

Hypermetabolism and weight loss have been previously shown among ALS patients [18]. Energy imbalance, altered thermogenesis, and gut microbiome alterations have been suggested as some of the mechanisms involved [18, 19]. Loss of appetite and dysphagia are two other factors suggested to contribute to weight loss among ALS patients [20]. Our findings on BMI are consistent with those of previous studies. A lower BMI has been associated with a higher risk for ALS [7, 11, 21–23]. We additionally showed that, during the decades before diagnosis, the rate of BMI increase tended to be different between ALS patients and controls. Although ALS patients had a higher BMI compared to controls earlier in life, they presented with lower BMI during the 10 years before diagnosis, in accordance with the previous studies [22, 23]. This finding was sustained after adjusting for age and sex. A study by Westeneng et al. suggested that the median BMI was higher among C9- patients but lower among C9 + patients, compared to controls, during several decades before diagnosis [7]. We showed a similar trend for C9- patients compared to controls as in the entire sample; however, the difference was less tangible between C9 + patients and controls. A lower BMI at diagnosis [21, 24–26] and an increased weight loss before diagnosis [27, 28] have been previously related to a higher risk of death after ALS diagnosis. Other studies that considered ALSFRS-R score as a measure of disease progression have also shown that a lower BMI is associated with a faster functional decline [29]. In line with prior studies, we showed that a higher BMI was associated with a lower risk of death among ALS patients, after accounting for the functional decline over time.

Besides BMI, another indication for metabolic dysregulation is altered lipid metabolism. The association observed for a history of hypercholesterolemia and a higher risk of ALS in this study is consistent with Mendelian randomization studies suggesting a causal positive association between total cholesterol and low-density lipoprotein (LDL) and ALS risk [30, 31] as well as with observational studies indicating a higher level of total cholesterol among ALS cases than controls [32]. Previous research by our group and others has found a null or inverse association between statin use and ALS risk [33, 34]. Thus, even if we assume that most participants reporting hypercholesterolemia had taken cholesterol-lowering medications, including statins, the observed association of hypercholesterolemia with ALS risk might not be entirely explained by medication use. The link between hypercholesterolemia and ALS is believably complex. Long-term hypercholesterolemia might be protective against ALS as lipids can serve as an alternative energy source for neurons, counteracting the neurodegenerative effects of energy deficit [35]. On the other hand, ALS-related pathologies might lead to alterations in cholesterol metabolism, potentially leading to the development of hypercholesterolemia. For instance, TDP-43 was previously shown to modify the expression of the sterol regulatory element binding transcription factor 2 (SREBF2) protein which is involved in the transcriptional control of cholesterol metabolism [36]. Further studies are therefore needed to better understand whether the higher-than-expected prevalence of hypercholesterolemia among patients with ALS is an upstream or secondary event of ALS. Our finding of an increased mortality among ALS patients with a history of hypercholesterolemia contrasts, on the other hand, with a prior study suggesting that higher lipoprotein levels measured at the time of diagnosis conveyed a lower risk of death after ALS diagnosis [37]. However, it is worth noting that we studied the history of hypercholesterolemia, whereas the previous study examined levels of lipid fractions, which were mostly within the normal range.

We found an inverse association between a history of diabetes mellitus and ALS risk, which is in line with previous research showing a protective role of type 2 diabetes in the risk of ALS [38–40]. Based on the information we had available from the Euro-MOTOR questionnaire we could not explicitly differentiate diabetes mellitus type 2 from type 1. Considering that diabetes mellitus type 1 has been shown to have a positive association with the risk of ALS, we expect that the misclassification of some cases of diabetes mellitus type 1 as type 2 would most likely have underestimated the real inverse association between diabetes type 2 and risk of ALS [38, 41]. Previous studies have suggested several pathways potentially underlying the observed inverse association between diabetes mellitus type 2 and ALS risk; for instance, a high circulating level of glucose and a higher energy balance can potentially counteract the energy deficit of ALS related to aberrant aggregations of proteins in neurons [35]. The observed association might also be in part attributed to the use of antidiabetic medication [42]. Nevertheless, the effect of antidiabetic medication was not possible to investigate as the Euro-MOTOR questionnaire does not ask for such information.

In our study, we found hypertension to be associated with both a higher risk of death as well as a faster functional decline in ALS patients. In line with our results, history of hypertension was previously linked to a higher risk of death among ALS patients [43, 44]. In contrast to our study, however, another study showed an inverse association of hypertension with ALS risk [45]. The contrasting findings might be because the increment in systolic and diastolic blood pressure might eventually have opposite effects on the risk for ALS; namely, systolic blood pressure might be a risk factor, whereas diastolic blood pressure might be a protective factor, for ALS [46]. In a sample of healthy individuals over 60 years, kidney function was found to negatively correlate with neurofilament light chain (NfL) [47], partially explaining the link between hypertension and a poorer prognosis after ALS diagnosis. A higher stage of chronic kidney disease and a lower concentration of cystatin C-based-eGFR have also been associated with ALS risk [48], indicating a potential explanatory mechanism for the link between hypertension and ALS.

Smoking has been considered for a long time as a potential risk factor for ALS. The exposure to oxidative stress and neurotoxic chemicals might provide a plausible biological explanation for this association [14]. Some observational studies showed a positive association for current smoking but no association for former smoking [7, 14, 15], while Mendelian Randomization studies have provided evidence for a causal link between smoking and risk for ALS [49]. Nevertheless, other studies, including a meta-analysis, suggested that smoking is not associated with the risk for ALS [11, 12] or risk of death after ALS diagnosis [13]. Our study did not find an association between current smoking and risk of ALS. Compared to controls, ALS patients might be more likely to quit smoking since the onset of symptoms, biasing therefore the association between smoking and ALS risk toward null. However, our study showed that current smoking and high level of smoking exposure (in pack-years) were associated with increased mortality risk among ALS patients, although the association was only statistically significant in the latter, in agreement with a previous study [50].

Head injury has been suggested as a risk factor for several neurodegenerative diseases as head trauma may for instance disrupt the permeability of the blood–brain barrier [51]. Additional indications for a role of head trauma in neurodegeneration include the TDP-43 depositions found in animal models following head trauma and in cases of chronic traumatic encephalopathy [51, 52]. A recent meta-analysis by Liu et al. [51] found a positive association between a history of head trauma and risk for ALS. Both severe and less severe head trauma were associated with an increased risk for ALS. The risk was three times as high for injuries occurring less than a year before diagnosis, indicating potentially reverse causation, considering that ALS is a disease with at least 1 year of diagnostic delay. The null result of the present study might also be partially attributed to a lack of statistical power to identify an association between a history of head injuries and the risk or prognosis of ALS. Further, as we had little information on the characteristics of head injuries, such as neuroimaging findings, we cannot preclude the possibility that head injuries of specific characteristics might still be related to the risk and prognosis of ALS.

The strengths of the present study lie in the inclusion of only incident ALS patients and the complete follow-up for all patients, alleviating concerns on selection bias due to recruitment of prevalent cases (i.e., patients with better survival than average) and loss to follow-up. A novelty of our study is the use of a study design that alleviates concern on familial confounding through analyzing discordant pairs of siblings or partners, because siblings and spouses share genetic or non-genetic factors that might be common causes of the studied lifestyle and medical conditions and ALS. The simultaneous investigation of the risk and prognosis of ALS, together with the exploration of the possible interplay between lifestyle and medical conditions and age, sex, and *C9orf72* mutation status, constitute two other novelties of the present study, compared to the existing literature. Finally, employing the joint model accounting for the longitudinal changes of ALSFRS-R, facilitated a more robust prognostic analysis [53].

Still, this study has several limitations. First, the information on exposure status was self-reported and obtained retrospectively. Thus, some degree of recall bias might exist for cases and the controls. For BMI during the decades before recruitment, it is possible that both cases and controls did not remember accurately, introducing some degree of non-differential misclassification. If this is the case, the effect we observed might have underestimated the real effect of BMI on ALS risk. Second, we had information on diabetes mellitus, hypercholesterolemia, and hypertension through self-reported diagnosis and on hypercholesterolemia and hypertension through also medication use; however, we had little information on how well the conditions were controlled. As a result, it remains to be examined whether the results would differ between conditions that are well controlled and conditions that are poorly controlled. Third, the sample size was not large enough to study these associations separately for sibling and partner controls. Further, we did not measure the extent of the *C9orf72* hexanucleotide extension for the ALS patients; as a result, it remains to be assessed whether the number of repeats could modify some of the noted associations in the study. Finally, although we studied a rather extensive list of factors potentially related to the risk and prognosis of ALS, we focused on the individual roles of these factors. Future studies are encouraged to examine the potentially synergistic effects of different risk factors. For instance, to better understand the link between BMI and ALS, other variables related to energy intake and expenditure, including dietary factors, physical activity, and the composition and functionality of gut microbiota should also be taken into account.

## Conclusion

Based on the Swedish ALSrisc Study, a higher BMI and previous diabetes mellitus were associated with a lower risk of ALS. In comparison, hypercholesterolemia was associated with a higher risk of ALS. A higher BMI was also associated with a lower risk of death, whereas smoking (especially in high pack-years), hypercholesterolemia, and hypertension were associated with a higher risk of death, after ALS diagnosis. Further investigation is warranted to understand whether the lower BMI, lower prevalence of diabetes mellitus, and higher prevalence of hypercholesterolemia among ALS patients are risk factors for ALS or secondary to ALS. The findings of a higher risk of death among ALS patients with smoking (especially heavy smoking), hypercholesterolemia, and hypertension underscore on the other hand the importance of targeting these conditions, with the aim of improving the survival of ALS patients.

### Supplementary Information

Below is the link to the electronic supplementary material.

Supplementary file1 (DOC 86 KB)

Supplementary file2 (DOCX 37 KB)

## Data Availability

Researchers can apply for access to data from the present study for well-defined research questions that are in line with the overall research agenda of the ALSrisc Study. For more information, please visit https://ki.se/en/cns/pre-clinical-research.
