# Supplementary material for: Lifestyle and medical conditions in relation to ALS risk and progression—an introduction to the Swedish ALSrisc Study
Source: J Neurol. 2024 Jun 15;271(8):5447–59. doi: 10.1007/s00415-024-12496-w (PMC11319377; doi:10.1007/s00415-024-12496-w)
Supplement: Supplementary file 2 — Supplementary file2 (DOCX 37 KB) [file 415_2024_12496_MOESM2_ESM.docx]

| Supplementary Table 1. Baseline characteristics of C9+ and C9- ALS patients | | |
| --- | --- | --- |
| **Characteristics** | **Cases C9-**  **(n=199)** | **Cases C9+**  **(n=22)** |
| **Age at diagnosis, mean (SD) [N]** | 65.07 (10.88) [199] | 61.54 (8.16) [22] |
| **Female, N (%)** | 91 (45.73) | 9 (40.91) |
| **BMI at diagnosis in Kg/m^2^, mean (SD) [N]** | 24.36 (3.59) [197] | 24.21 (3.51) [22] |
| **BMI during the 10 years before diagnosis, mean (SD) [N]** | 25.67 (4.06) [184] | 25.75 (3.24) [22] |
| **BMI during the 20 years before diagnosis, mean (SD) [N]** | 25.15 (4.00) [175] | 24.83 (3.73) [22] |
| **BMI during the 30 years before diagnosis, mean (SD) [N]** | 23.82 (3.24) [158] | 22.62 (1.98) [21] |
| **BMI during the 40 years before diagnosis, mean (SD) [N]** | 22.95 (3.76) [138] | 22.08 (1.99) [17] |
| **Smoking status, N (%)** |  |  |
| Current smokers | 14 (7.04) | 3 (13.64) |
| Former smokers | 89 (44.72) | 8 (36.36) |
| Never smoked | 96 (48.24) | 11 (50.0) |
| **Smoking in pack-years, N (%)** |  |  |
| <7 | 24 (12.06) | 4 (18.18) |
| 7-16 | 27 (13.57) | 1 (4.55) |
| >16 | 34 (17.09) | 3 (13.64) |
| Never smoked | 96 (48.24) | 11 (50.0) |
| **History of head injuries, N (%)** |  |  |
| Yes | 37 (18.59) | 3 (13.64) |
| No | 162 (81.41) | 19 (86.36) |
| **History of diabetes mellitus, N (%)** |  |  |
| Yes | 14 (7.18) | 1 (4.55) |
| No | 181 (92.82) | 21 (95.45) |
| **History of hypertension, N (%)** |  |  |
| Yes | 68 (36.96) | 7 (33.33) |
| No | 116 (63.04) | 14 (66.67) |
| **History of hypercholesterolemia, N (%)** |  |  |
| Yes | 47 (24.23) | 6 (27.27) |
| No | 147 (75.77) | 16 (72.73) |
| **ALSFRS-R at diagnosis, mean (SD) [N]** | 38.26 (6.92) [168] | 37.63 (7.43) [19] |
| **Progression rate at diagnosis, median (IQR) [N]** | 0.56 (0.29-1.06) [165] | 0.89 (0.34-1.70) [19] |
| **Diagnostic delay, median (IQR) [N]** | 13.81 (8.89-22.26) [196] | 11.04 (7.06-15.18) [22] |
| **Onset site, N (%)** |  |  |
| Other | 13 (6.53) | 1 (4.55) |
| Spinal | 128 (64.32) | 10 (45.45) |
| Bulbar | 56 (28.14) | 11 (50.00) |
| Missing | 2 (1.01) | 0 (0.0) |
| ALS: amyotrophic lateral sclerosis, BMI: body mass index, IQR: interquartile range, SD: standard deviation, C9+/-: patients with or without the *C9orf72* mutation | | |

| Supplementary Table 2. Adjusted hazard ratio (HR) with 95% confidence interval (CI) for risk of death after ALS diagnosis in relation to BMI, smoking, and history of head injuries, diabetes mellitus, hypercholesterolemia, or hypertension using Cox model | | |
| --- | --- | --- |
| **Characteristics** | N of events (IR) | HR (95% CI) |
| **BMI at diagnosis (per 1 Kg/m^2^)** | 151 (0.34) | 0.97 (0.92, 1.02) |
| **BMI during the 10 years before diagnosis (per 1 Kg/m^2^)** | 143 (0.34) | 0.98 (0.93, 1.02) |
| **BMI during the 20 years before diagnosis (per 1 Kg/m^2^)** | 136 (0.34) | 0.99 (0.94, 1.03) |
| **BMI during the 30 years before diagnosis (per 1 Kg/m^2^)** | 126 (0.35) | 0.97 (0.91, 1.03) |
| **BMI during the 40 years before diagnosis (per 1 Kg/m^2^)** | 113 (0.37) | 0.96 (0.90, 1.03) |
| **Smoking status** |  |  |
| Current smokers | 13 (0.38) | 1.48 (0.80, 2.73) |
| Former smokers | 72 (0.42) | 1.43 (1.00, 2.03) |
| Never smoked | 66 (0.28) | Ref |
| **Smoking in pack-years** |  |  |
| <7 | 18 (0.36) | 1.44 (0.84, 2.46) |
| 7-16 | 27 (0.42) | 1.43 (0.88, 2.31) |
| >16 | 28 (0.56) | 1.82 (1.15, 2.91) |
| Never smoked | 66 (0.28) | Ref |
| **History of head injuries** |  |  |
| Yes | 23 (0.39) | 1.16 (0.73, 1.84) |
| No | 128 (0.34) | Ref |
| **History of diabetes mellitus** |  |  |
| Yes | 10 (0.29) | 0.68 (0.35, 1.32) |
| No | 137 (0.34) | Ref |
| **History of hypercholesterolemia** |  |  |
| Yes | 42 (0.40) | 1.09 (0.76, 1.56) |
| No | 105 (0.32) | Ref |
| **History of hypertension** |  |  |
| Yes | 62 (0.46) | **1.45 (1.02, 2.07)** |
| No | 74 (0.27) | Ref |
| ALS: amyotrophic lateral sclerosis, BMI: body mass index, IR: incidence rate, Ref: reference | | |

| Supplementary Table 3. Average change in ALSFRS-R score over time after ALS diagnosis in relation to BMI, smoking, and history of head injuries, diabetes mellitus, hypercholesterolemia, or hypertension using linear mixed models with random intercept and slope | |
| --- | --- |
| **Characteristics** | β (95%CI) |
| **BMI at diagnosis (per 1 Kg/m^2^)** | -0.12 (-0.49, 0.25) |
| **BMI during the 10 years before diagnosis (per 1 Kg/m^2^)** | **-0.50 (-0.80, -0.21)** |
| **BMI during the 20 years before diagnosis (per 1 Kg/m^2^)** | -0.25 (-0.53, 0.03) |
| **BMI during the 30 years before diagnosis (per 1 Kg/m^2^)** | -0.30 (-0.77, 0.17) |
| **BMI during the 40 years before diagnosis (per 1 Kg/m^2^)** | 0.07 (-0.37, 0.50) |
| **Smoking status** |  |
| Current smokers | 3.15 (-2.24, 8.54) |
| Former smokers | -0.91 (-3.62, 1.79) |
| Never smoked | Ref |
| **Smoking in pack-years** |  |
| <7 | 0.20 (-3.62, 4.03) |
| 7-16 | -2.80 (-6.11, 0.50) |
| >16 | **-3.81 (-7.24, -0.38)** |
| Never smoked | Ref |
| **History of head injuries** |  |
| Yes | -3.09 (-6.54, 0.36) |
| No | Ref |
| **History of diabetes mellitus** |  |
| Yes | 0.40 (-4.25, 5.05) |
| No | Ref |
| **History of hypercholesterolemia** |  |
| Yes | -2.86 (-5.75, 0.02) |
| No | Ref |
| **History of hypertension** |  |
| Yes | **-4.55 (-7.22, -1.89)** |
| No | Ref |
| ALS: amyotrophic lateral sclerosis, ALSFRS-R: amyotrophic lateral sclerosis functional rating scale-revised, BMI: body mass index, CI: confidence interval, Ref: reference | |
